# The Role of High Frequency Ultrasonography in Diagnosis of Acute Closed Mallet Finger Injury

**DOI:** 10.1038/s41598-017-10959-x

**Published:** 2017-09-08

**Authors:** Tiezheng Wang, Hengtao Qi, Jianbo Teng, Zengtao Wang, Bin Zhao

**Affiliations:** 1 Shandong University, Shandong Medical Imaging Research Institute, Shandong, China; 2 Provincial Hospital Affiliated to Shandong University, Shandong, China

## Abstract

To evaluate the role of high frequency ultrasonography in diagnosis of acute closed mallet finger injury. 36 patients diagnosed with mallet finger were included in this study. All patients underwent ultrasonography, magnetic resonance imaging(MRI) and X-ray examinations. A new kind of classification of acute mallet finger injury based on ultrasonography findings was described. The difference in terms of extensor tendon injury and bony fragment identification ability among the three types of examinations were described respectively. Either an injury of extensor digital tendon or an avulsion fracture of distal phalangeal base was identified clearly on ultrasonography. Among the 36 cases, avulsion fracture of the distal phalangeal base was found without extensor tendon rupture in *Type A*, complete rupture of the extensor tendon was found without avulsion fracture in *Type B*, and contusion of the extensor tendon was found in *Type C*. Compared with X-ray, ultrasonography and MRI could show the extensor tendon injury clearly. While compared with MRI, ultrasonography and X-ray was more sensitive in showing bony fragment. High frequency ultrasonography could be an accurate, reliable, and non-invasive diagnostic imaging method in diagnosis of acute close mallet finger injury.

## Introduction

Mallet finger injury presents with a flexion deformity of the distal interphalageal joint (DIP) caused by disruption of the extensor mechanism and loss of extension power consequently^1–3^. A clear history like minor trauma during playing basketball or house work has been reported as a common etiology^4, 5^. At present, orthopedic diagnosis of acute close mallet finger injury is mainly based on clinical signs, symptoms and X-ray examination outcomes. However, X-ray examination can only give us information of whether or not there is a bony injury. And it is well known that objective evaluation of the extensor tendon status need to be evaluated on either high frequency ultrasonography or MRI, which has been seldom done according to literature^6–8^.

The purpose of this study was to observe the clinical application and usage of high frequency ultrasonography in the diagnosis of acute closed mallet finger injury, compared to that of X-ray and MRI examination.

## Material and Methods

Thirty-six patients each with an acute closed mallet finger injury (age range: 12–56 years; mean age: 32.2 years) referred to the orthopaedic department of our hospital from Sep 2009 to Jul 2015 were included into this study. The study protocol was approved by the ethics committee of Shandong Medical Imaging Research Institute and all methods were performed in accordance with the relevant guidelines and regulations. All participants provided written informed consent. All presented with a flexion deformity of a single finger at the distal interphalangeal joint (DIP). Duration of symptoms reported by the patients varied from 1 day to 2 weeks. Detailed clinical and imaging examination data was summarized in Table 1.

**Table 1:** The detailed clinical profiles and image examination for mallet finger.

Patient no./sex./age (years)	Finger affected	Duration of symptoms	Imaging examination
1/M/23	Right forefinger	Two days	US, X-ray, MRI
2/M/35	Left middle finger	One week	US, X-ray, MRI
3/M/18	Right forefinger	One day	US, X-ray, MRI
4/M/27	Right ring finger	Two days	US, X-ray, MRI
5/M/27	Left middle finger	Two days	US, X-ray, MRI
6/F/21	Right ring finger	Three days	US, X-ray, MRI
7/M/35	Right forefinger	One week	US, X-ray, MRI
8/M/31	Right middle finger	Five days	US, X-ray, MRI
9/M/44	Right ring finger	Four days	US, X-ray, MRI
10/F/27	Right middle finger	One week	US, X-ray, MRI
11/M/50	Left forefinger	Two weeks	US, X-ray, MRI
12/M/17	Right ring finger	Two days	US, X-ray, MRI
13/M/31	Right ring finger	Four days	US, X-ray, MRI
14/M/13	Right middle finger	Three days	US, X-ray, MRI
15/F/45	Right middle finger	One week	US, X-ray, MRI
16/F/51	Right ring finger	Five days	US, X-ray, MRI
17/M/22	Left ring finger	Four days	US, X-ray, MRI
18/M/12	Right middle finger	One week	US, X-ray, MRI
19/M/56	right forefinger	Two weeks	US, X-ray, MRI
20/M/30	Right little finger	Two days	US, X-ray, MRI
21/M/33	Right middle finger	One day	US, X-ray, MRI
22/F/47	Right ring finger	Two weeks	US, X-ray, MRI
23/M/18	Right middle finger	Three days	US, X-ray, MRI
24/M/29	Right little finger	One week	US, X-ray, MRI
25/M/43	right forefinger	Five days	US, X-ray, MRI
26/M/47	Right ring finger	Four days	US, X-ray, MRI
27/M/54	Right ring finger	Two weeks	US, X-ray, MRI
28/M/34	Right middle finger	Two days	US, X-ray, MRI
29/M/46	Left little finger	Four days	US, X-ray, MRI
30/M/31	Right middle finger	Three days	US, X-ray, MRI
31/F/26	Right ring finger	One week	US, X-ray, MRI
32/M/30	Right middle finger	Five days	US, X-ray, MRI
33/M/19	Left forefinger	Four days	US, X-ray, MRI
34/M/18	Right middle finger	Four days	US, X-ray, MRI
35/M/28	Right little finger	Three days	US, X-ray, MRI
36/M/41	Right ring finger	One week	US, X-ray, MRI

All patients underwent ultrasonography, X-ray, and MRI examinations. All the ultrasound examination was performed by an experienced cardiovascular ultrasound expert with more than 7 years of experience in cardiovascular ultrasound examination. Ultrasonography, X-ray and MRI images of the 36 cases were all assessed by two musculoskeletal radiologists blinded to the clinical diagnosis independently, with more than10 years of imaging diagnosing experience in interpreting X-ray, ultrasound and MRI examination results. Reference to original imaging reports was shielded to avoid bias.

A GE Vivid7 ultrasound system (GE healthcare, Holten Norway) with a 14 MHz broadband linear array probe (GE healthcare, Holten Norway) was used for ultrasonography examination. During the examination, a thick layer of gel or a small water sac was applied for better observation result. Both a static and a dynamic ultrasonography examination with longitudinal and axial sonograms were performed on the injured finger focusing of the extensor tendon insertion site on the base of the distal phalangeal bone. The static examination was performed with the injured finger in neutral position, while the dynamic examination was carried out by intended active and passive moving DIP joint and observing the real-time imaging of the extensor system, with a comparison with the contralateral healthy finger for the purpose of control.

Besides of observing the digital extensor tendon, base of the distal phalanx was carefully checked during the ultrasonography examination, as well as on X-ray and MRI images to exclude fracture. Non-contrast MRI images of T1WI and T2WI sequences in both axial and longitudinal plane were obtained using a 3.0 Tesla MRI system(GE Signa EXCITE HD 3.0 T, The USA) with a dedicated surface coil on the injured finger and the contralateral healthy finger, for the purpose of control. The ultrasonography, X-ray and MRI results of each case were summarized in Table 2 and Table 3.

**Table 2:** The image examination results of acute closed mallet finger (radiologist A).

	US (36 cases)	X-ray(36 cases)	MR(36 cases)
Avulsion fracture without extensor tendon rupture	9	9	9
Complete tendon rupture without fracture	23	—	22
Contusion of extensor tendons	4	—	5

**Table 3:** The image examination results of acute closed mallet finger (radiologist B).

	US (36 cases)	X-ray(36 cases)	MR(36 cases)
Avulsion fracture without extensor tendon rupture	9	9	9
Complete tendon rupture without fracture	22	—	22
Contusion of extensor tendons	5	—	5

For evaluation of the extensor system injury, a new ultrasonography classification method of acute closed mallet finger injury was proposed in this study according to the type and location of the injury (extensor tendon injury or fracture of the distal phalanx):*Type A*, avulsion fracture without extensor tendon rupture. *Type B*, complete tendon rupture without fracture. *Type C*, contusion of extensor tendons.

And for the other hand, in order to identify the difference in terms of extensor tendon injury identification ability among the three examinations, the images were classified into four grades according to the tendon and border with surrounding tissue appearance. Grade 0: the injured extensor tendon could not be identified. Grade 1: the injured extensor tendon was showed with poor detail. Grade 2: relatively clear identification of the injured extensor tendon quality. Grade 3: any slightly thin fiber bundles of the injured extensor tendon was visible with a good detail. Corresponding injured extensor tendon evaluation results were summarized in Table 4 and Table 5.

**Table 4:** The visibility of extensor tendon in the three examinations (radiologist A).

	Grade 0	Grade 1	Grade 2	Grade 3
US	0	1	10	25
MRI	0	1	8	27
X-ray	36	0	0	0

χ^2^ = (84.33), χ^2^ > χ^2^_0.05,2_ (5.99); *P* < 0.05.

**Table 5:** The visibility of extensor tendon in the three examinations (radiologist B).

	Grade 0	Grade 1	Grade 2	Grade 3
US	0	1	10	25
MRI	0	1	9	26
X-ray	36	0	0	0

χ^2^ = (83.27), χ^2^ > χ^2^_0.05,2_ (5.99); *P* < 0.05.

In addition, in order to identify the difference in terms of bony fragment identification ability among the three examinations, the images of 9 cases with avulsion fracture of the distal phalangeal base were classified into another four grades according to the bony fragment border with surrounding tissue appearance as well. Grade 0: bony fragment could not be identified. Grade 1: bony fragment was found with poor detail. Grade 2: bony fragment was relatively clear. Grade 3: a bony fragment was clearly visualized with a good detail. All bony fragment evaluation results were summarized in Table 6 and Table 7.

**Table 6:** The visibility of fracture fragment in the three examinations (radiologist A).

	Grade 0	Grade 1	Grade 2	Grade 3
US	0	1	1	7
MRI	0	5	3	1
X-ray	0	0	2	7

χ^2^ = (11.83), χ^2^ > χ^2^_0.05,2_ (5.99); *P* < 0.05

**Table 7:** The visibility of fracture fragment in the three examinations (radiologist B).

	Grade 0	Grade 1	Grade 2	Grade 3
US	0	1	1	7
MRI	0	4	4	1
X-ray	0	0	2	7

χ^2^ = (11.08), χ^2^ > χ^2^_0.05,2_ (5.99); *P* < 0.05.

The SPSS program (version 13.0, SPSS, Chicago, IL, USA) was used for statistical analysis. Kappa test was adopted to evaluate the inter-rater reliability for the original classifications. Kruskal-Wallis test was adopted to find statistical difference between identification ability of extensor tendon injury among the three examinations and difference between identification ability of bony fragment among the three examinations, and the Bonferroni method for multiple comparisons (α′ = 0.05/[3(3-1)/2] = 0.017). Paired t-test was used to find statistical difference of the average measured diameter of the extensor tendon between acute closed mallet fingers and contralateral fingers.

## Results

**Figure 1:**
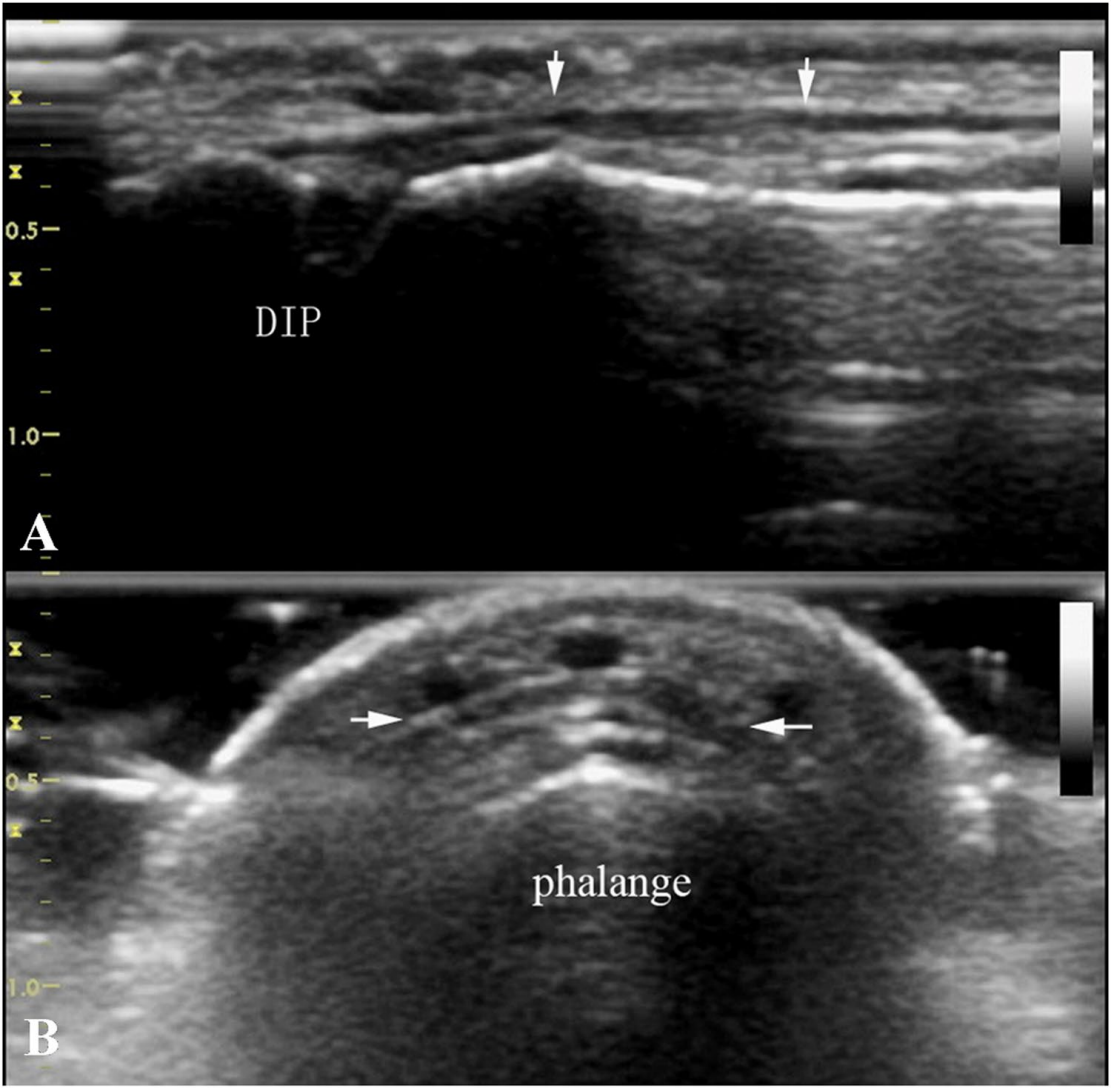
Normal extensor tendon of finger. High frequency ultrasonography image of extensor tendon in longitudinal plane (Fig. 1A) and transverse plane (Fig. 1B).

Normal finger extensor tendons appeared as slightly hyperechoic thin fiber bundles in longitudinal and transverse planes on ultrasonography^9, 10^. As shown in Fig. 1, the end of extensor tendon is attached to the base of the distal phalanx, and their integrity could be evaluated by sliding dynamic examination.

**Figure 2:**
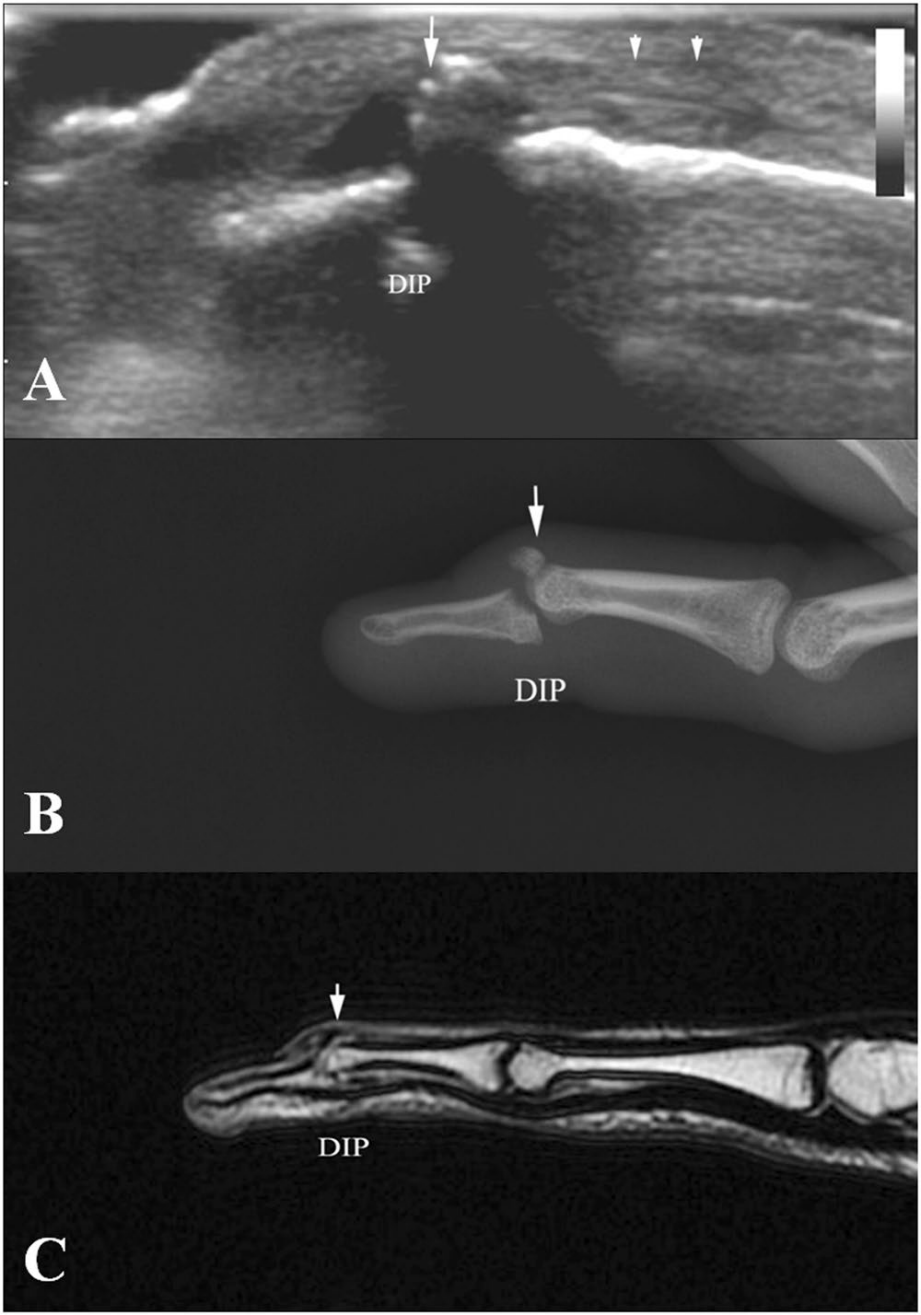
Type A mallet finger. High frequency ultrasonography, X-ray and MR images of type A mallet finger inury. Figure 2A: The retracted extensor tendon (short arrow), the avulsion bony fragment (long arrow); Fig. 2B and Fig. 2C: The avulsion bony fragment (arrow).

**Figure 3:**
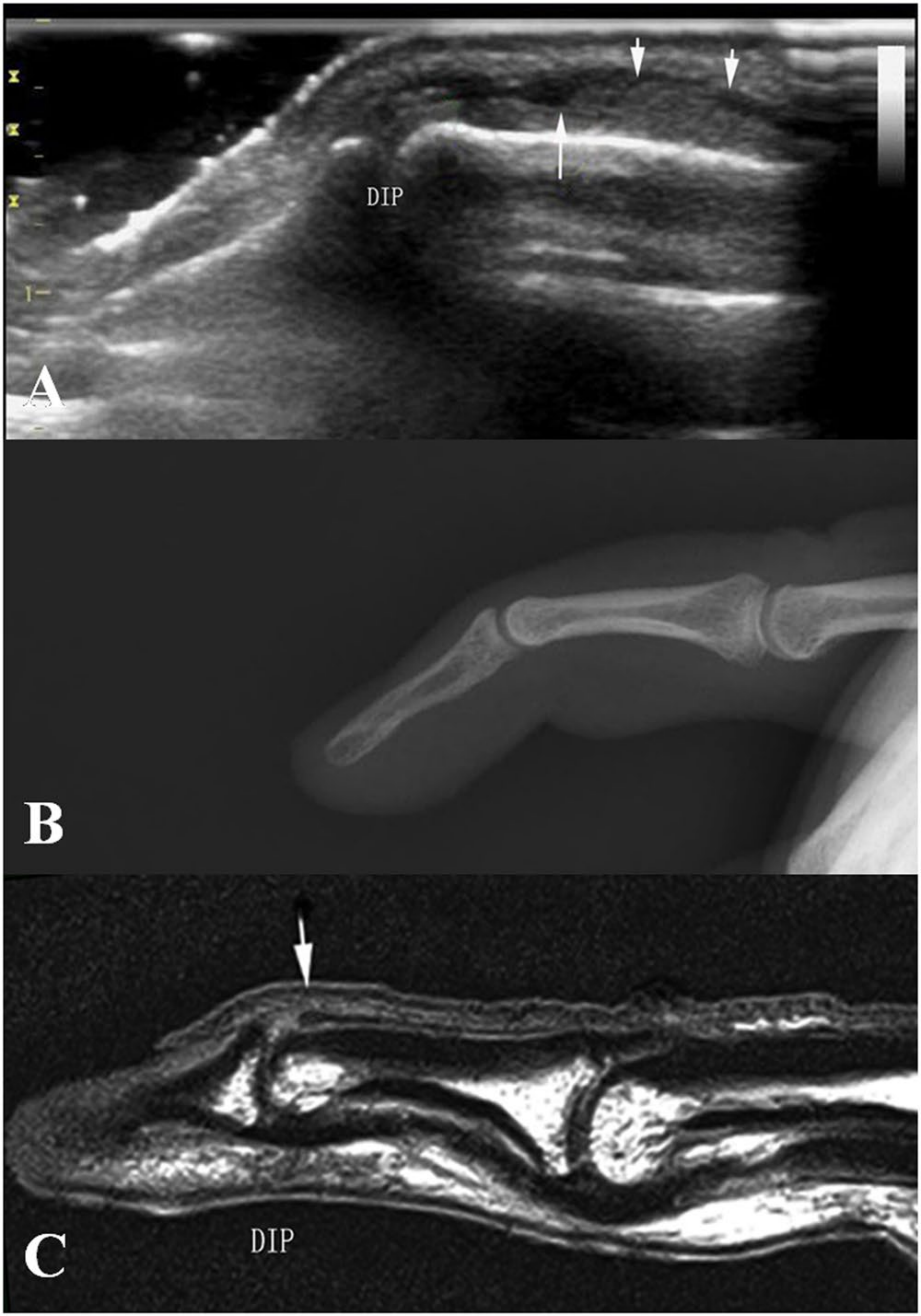
Type B mallet finger injury. High frequency ultrasonography, X-ray and MRI images of type B mallet finger. Figure 3A: The retracted extensor tendon (short arrow), the distal end of extensor tendon (long arrow); Fig. 3B: X-ray image of mallet finger injury. Figure 3C: the retracted extensor tendon (arrow).

**Figure 4:**
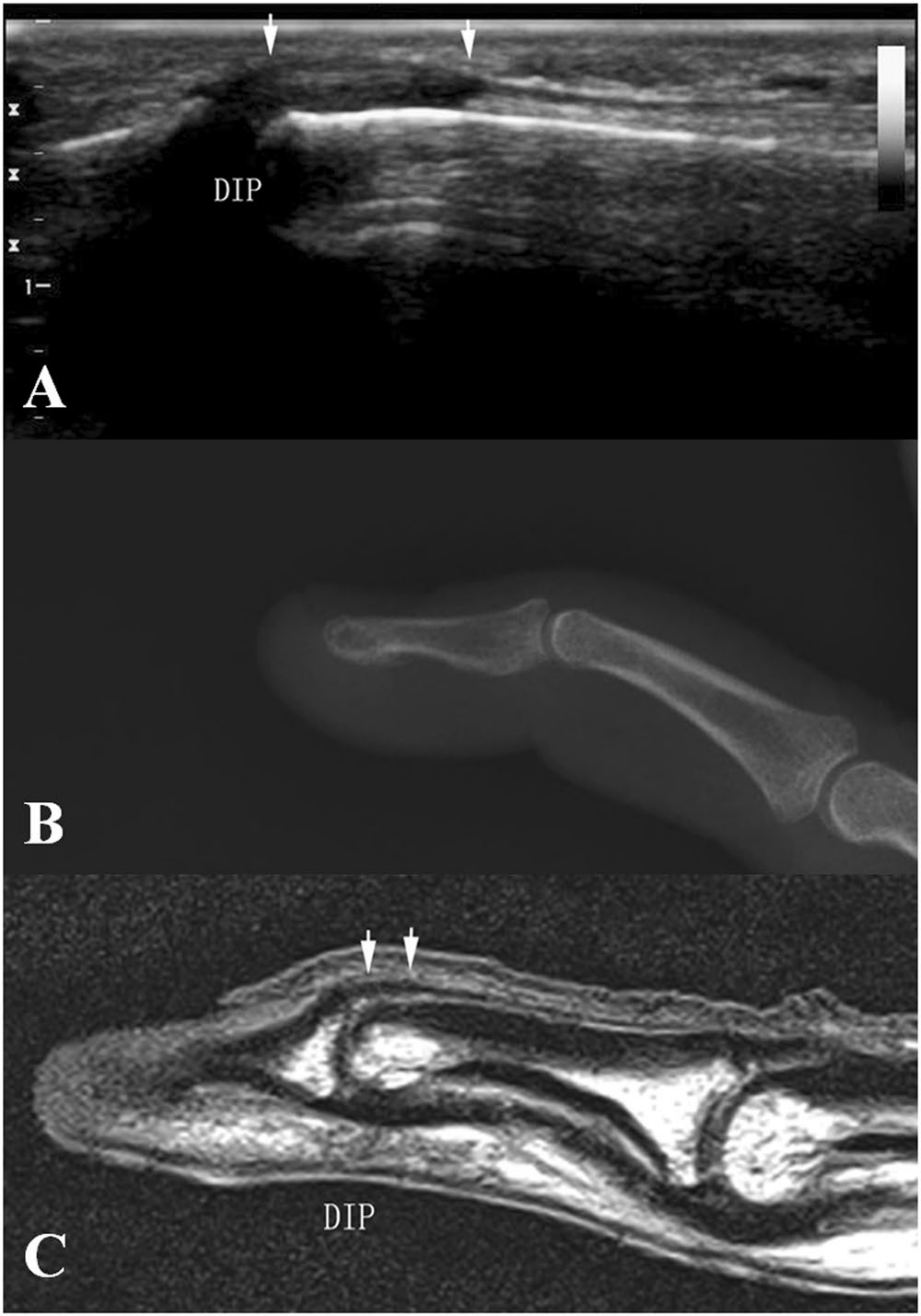
Type C mallet finger. High frequency ultrasonography (Fig. 4A), X-ray (Fig. 4B) and MRI (Fig. 4C) images of type C mallet finger injury. The injured extensor tendon (arrow).

According to ultrasonography findings of the present study, we divided acute closed mallet finger injury into three subtypes: *Type A*, avulsion fracture without extensor tendon rupture. The ultrasonography showed hyperechoic fracture fragments of the distal phalangeal base and the thicker extensor tendon due to shortening, with the hyperechoic fracture fragments at the distal margin. (Fig. 2); no real-time gliding of the extensor tendon was found during either active or passive movements of the DIP. *Type B*, complete tendon rupture without fracture. Longitudinal evaluation showed, in Fig. 3, the disruption of the extensor tendon at the level of DIPs with retraction of the proximal tendon stump but no fracture fragments of the distal phalangeal base; no real-time gliding of the extensor tendon during either active or passive movements of the DIP was found. *Type C*, contusion of extensor tendons. The ultrasonography showed the thicker and hypoechoic extensor tendon which was still integrate in longitudinal plane (Fig. 4). Real-time gliding of the extensor tendon during both active and passive movements of the DIP could be found. The homogeneity for the classifications was high between two radiologists (κ = 0.948).

In evaluating the extensor tendon injury, most findings of MRI examination were in line with that of the ultrasonography. For example, in type A and B, longitudinal MRI images showed retraction of the extensor tendon as clearly as ultrasonography. Same in type C, MRI images showed as clearly as ultrasonography that although the injured extensor tendon was thicker and had lower signals on T1-weighted images when compared with the contralateral healthy one, while in longitudinal plane, its integrity was still continuous. In the same time, X-ray could not show the extensor tendon clearly. Significant difference was found among all three examinations (*p* < 0.05), with even highest significance when taken separately, ultrasonography and X-ray, MRI and X-ray respectively (*p* < 0.017). We found no significant difference between ultrasonography and MRI (*p* = 0.613, 0.803, *p* > 0.017).

For assessing avulsion fracture of the distal phalangeal base, most findings of the ultrasonography results were in line with the X-ray findings in type A. Fracture fragment could be clearly shown as a hyperechoic or hyperintense fragment with good contrast. However MRI was not able to demonstrate the fracture fragment clearly in most cases of this study. Significant difference was found among all three examinations (*p* < 0.05), with even highest significance when taken separately, ultrasonography and MRI, X-ray and MRI (*p* < 0.017) respectively. We found no significant difference between ultrasonography and X-ray examination (*p* = 0.903, 0.903, *p* > 0.017).

Average diameter of the extensor tendon at the distal end in acute closed mallet finger injuries measured by ultrasonography was 0.16 ± 0.05 cm in 36 patients, while in comparison, the average diameter of that on the similar site in the contralateral fingers was 0.12 ± 0.03 cm. The difference was statistically significant (*t* = 7.67, *p* < 0.05). And the injured extensor tendons were thicker in all the three types.

For treatment, 5 out of 36 patients (3 type A, 2 type B) underwent open surgical repair either because in some type A cases the avulsion fracture was an intra-articular one with a bone fragment larger than 1/3 of the DIP articular area which will influence DIP joint function, or because in some type B cases the proximal extensor tendon stump retracted far back from the base of the distal phalanx on a distance higher than 4.0 mm, which according to our clinical experience makes conservative treatment difficult. Moreover, the intraoperative clinical findings of those five cases were in good accord with preoperative ultrasonography examination results. The rest 31 patients adopted conservation treatments with splint fixation. Follow-up of the patients confirmed a good outcome with satisfying results in both surgically and conservatively treated cases.

## Discussion

Acute closed mallet finger injury is a common hand injury caused by disruption of the extensor mechanism. Most cases are due to abrupt flexion of an actively extending distal phalanx, such as hitting finger tips when attempting to catch a flying basketball, while some other milder injuries could happen during minor actions such as lifting pants or fastening buttons.

Literature report on imaging methods evaluation of mallet finger injury was rare. As we all know, X-ray exanimation can demonstrate bony injury directly and tendinous injury indirectly with reference to clinical examination results. With technical development of high frequency ultrasound and MRI, their application in the musculoskeletal system injury diagnosis has been extensively broadened. This study proved that both ultrasonography and MRI were useful in the diagnosis of mallet finger injury with high accuracy, reliability, and safety. In type B, ultrasonography can show the disruption of the extensor tendon with retraction of the proximal tendon stump without avulsion fracture clearly; in type C, ultrasonography can show that the extensor tendons were thicker and hypoechoic which were still integrate in longitudinal plane. In bony mallet fingers(type A), although ultrasonography is inferior to X-ray and MRI in demonstrating a volar subluxation of distal phalanx, ultrasonography was more sensitive and accurate in showing bony fragment compared to MRI. More importantly, compared to MRI, high frequency ultrasonography allows evaluation of real-time function of the extensor tendon through active or passive DIP joint movements. Furthermore, as we all know compared to MRI, ultrasonography was a quicker, more affordable, and less stressing screening choice^11–14^. So we can observe the thickness change of injured extensor tendon after treatment by ultrasonography, which is also an important aspect in the following-up^15, 16^. As time goes on, the thickness of injured extensor tendons could decrease gradually with the swelling lightening.

Mallet finger injury often needs to be distinguished from psoriatic arthritis and rheumatoid arthritis in clinical differential diagnosis. In patients with psoriatic arthritis or rheumatoid arthritis, the extensor tendon is continuous, and its sliding range is normal during passive movements of the DIP under ultrasonography. In mallet finger injury cases, however, disruption and thickening of extensor tendons are present, and sliding action of the extensor tendon reduces or even disappears during passive movements of the DIP under ultrasonography. Those above image characters can be used to differentiate mallet finger from rheumatoid arthritis. And more importantly, we can differentiate mallet finger injury from psoriatic arthritis or rheumatoid arthritis by medical history and laboratory examination easily.

Regarding treatment of acute closed mallet finger injury, clinical physicians recommend either conservative or surgical treatment based on the type and extent of the injury. Surgery should be adopted either because in some type A cases the avulsion fracture was an intra-articular one with a bone fragment larger than 1/3 of the DIP articular area which will influence DIP joint function, or because in some type B cases the proximal extensor tendon stump retracted far back from the base of the distal phalanx on a distance higher than 4.0 mm, which according to our clinical experience makes conservative treatment difficult. For other conditions, conservative treatment with external splint fixation for 6 to 8 weeks would be more appropriate. Satisfactory outcome was confirmed in this study during clinical follow-up visits in both surgically and conservatively treated cases^17–19^.

This study did have some limitations. First, the sample size is small, and the sample size should be expanded in order to get more concrete conclusions. Second, although ultrasonography can show some bony structural abnormalities, it would be difficult to detect a volar subluxation of distal phalanx in bony mallet finger with dorsal examination, which could be detected by X-ray and MRI. This is a limitation of ultrasonography assessments in diagnosis of mallet fingers.

## Conclusion

High frequency ultrasonography can be used as an accurate, reliable, and non invasive diagnostic imaging method in the assessment of both bony and tendinous mallet finger injury. And more importantly, real-time ultrasonography can help with evaluating function of the extensor tendon. Therefore, it can provide a beneficial guidance to clinical diagnosis and treatment. Further research to increase its clinical application in mallet finger injury diagnosis and treatment should be continued.
